# Reduced Regional Grey Matter Volumes in Pediatric Obstructive Sleep Apnea

**DOI:** 10.1038/srep44566

**Published:** 2017-03-17

**Authors:** Mona F. Philby, Paul M. Macey, Richard A. Ma, Rajesh Kumar, David Gozal, Leila Kheirandish-Gozal

**Affiliations:** 1 Section of Pediatric Sleep Medicine, Department of Pediatrics, Pritzker School of Medicine, Biological Sciences Division, The University of Chicago, Chicago, IL, USA; 2 UCLA School of Nursing University of California at Los Angeles, Los Angeles, CA 90095, USA; 3 The Brain Research Institute, University of California at Los Angeles, Los Angeles, CA 90095, USA; 4 Department of Anesthesiology, University of California at Los Angeles, Los Angeles, CA 90095, USA; 5 Department of Bioengineering, University of California at Los Angeles, Los Angeles, CA 90095, USA; 6 Department of Radiological Sciences, University of California at Los Angeles, Los Angeles, CA 90095, USA.

## Abstract

Pediatric OSA is associated with cognitive risk. Since adult OSA manifests MRI evidence of brain injury, and animal models lead to regional neuronal losses, pediatric OSA patients may also be affected. We assessed the presence of neuronal injury, measured as regional grey matter volume, in 16 OSA children (8 male, 8.1 ± 2.2 years, AHI:11.1 ± 5.9 events/hr), and 200 control subjects (84 male, 8.2 ± 2.0 years), 191 of whom were from the NIH-Pediatric MRI database. High resolution T1-weighted whole-brain images were assessed between groups with voxel-based morphometry, using ANCOVA (covariates, age and gender; family-wise error correction, *P* < 0.01). Significant grey matter volume reductions appeared in OSA throughout areas of the superior frontal and prefrontal, and superior and lateral parietal cortices. Other affected sites included the brainstem, ventral medial prefrontal cortex, and superior temporal lobe, mostly on the left side. Thus, pediatric OSA subjects show extensive regionally-demarcated grey matter volume reductions in areas that control cognition and mood functions, even if such losses are apparently independent of cognitive deficits. Since OSA disease duration in our subjects is unknown, these findings may result from either delayed neuronal development, neuronal damaging processes, or a combination thereof, and could either reflect neuronal atrophy or reductions in cellular volume (neurons and glia).

Obstructive sleep apnea (OSA) in children is highly prevalent, being reported to affect up to 5% of all children^1^,^2^. The condition consists in the development of increased upper airway resistance, alveolar hypoventilation, and episodic upper airway collapse during sleep. These recurrent events can be associated with both episodic hypoxemia and arousals, following which children will resume sleep, thereby resulting in fragmented and non-restorative sleep^2^.

One of the major consequences of pediatric OSA consists in the increased risk for severity-dependent cognitive and behavioral deficits, and poor school performance^3^,^4^,^5^,^6^,^7^,^8^,^9^,^10^,^11^. In the majority of published intervention studies, effective OSA treatment has led to either significant improvements or to restoration of cognitive performance^12^,^13^,^14^. However, examination of anatomical brain changes in children with OSA that may contribute to reported cognitive deficits has not been thoroughly explored. Recently, we reported functional MRI findings in a subgroup of children with mild OSA who did not manifest an evidence of cognitive deficits on the enhanced regional recruitment of specific brain regions during an attention-executive task^15^, but this study did not explicitly explore evidence for regional grey matter losses.

Earlier animal studies from our laboratory clearly showed that intermittent hypoxia and sleep fragmentation, primary characteristics of OSA, induce discernible neuronal cell losses in several brain regions^16^. Furthermore, the developing brain appears to be exquisitely more vulnerable to such perturbations^17^. In addition, in multiple MR imaging studies in adult OSA patients, the presence of significant neural and axonal injury has been inferred along with their potential reversibility with treatment^18^,^19^,^20^,^21^,^22^,^23^,^24^.

Based on aforementioned considerations, the present study aimed to assess the presence of potential injury to neuronal areas measured as reduced grey matter volume in a group of pediatric OSA patients relative to a large set of comparable control subjects.

## Methods

### Subjects

The study was approved by the human subject committee at the University of Chicago (IRB Protocol # 11-0280-CR004), and informed consent was obtained from the legal caregiver of each participant. Assent was obtained from children >7 years old. Consecutive children being evaluated for habitual snoring, who were diagnosed as OSA with overnight polysomnography, were invited to participate between October 2014 and September 2015. Controls were recruited through our well-child clinics, after ascertaining that they did not snore based on a validated questionnaire, and further subjected to a sleep study to confirm the absence of sleep disordered breathing. Participants underwent baseline anthropometric assessments, as well as overnight polysomnography, which were interpreted using standard criteria. In addition, a neurocognitive battery was also administered to participants in the morning (starting at 9:00 AM) after breakfast and following the sleep study. All methods were performed in accordance with the relevant guidelines and regulations.

### Exclusion Criteria

Children with diagnosed ADHD or using psychostimulant medications (n = 5), as well as with known neurodevelopmental delays (n = 1) were excluded. In addition, children with hypertension or using anti-hypertensive drug therapies were excluded (n = 2). Furthermore, children with either known or suspected diabetes, as delineated by the Global IDF/ISPAD Guideline for Diabetes in Childhood and Adolescence (http://www.idf.org/sites/default/files/Diabetes-in-Childhood-and-Adolescence-Guidelines.pdf; n = 1), with a craniofacial, neuromuscular or defined genetic syndrome, and children on chronic anti-inflammatory therapy (n = 1), or with any known acute or chronic illness were also excluded.

### Anthropometry

Height and weight centiles were calculated using the Centre for Disease Control 2000 and body mass index (BMI) z-scores were calculated using The Children’s Hospital of Philadelphia online software (http://stokes.chop.edu/web/zscore). A BMI z-score >1.65 was considered as obesity.

### Sphygmomanometry

All children had arterial blood pressure measured. Systolic and diastolic BP indices (SBPi and DBPi, respectively) were calculated by dividing the average systolic and diastolic pressure by the respective 95th percentile for BP using National Heart, Lung and Blood Institute guidelines (www.nhlbi.nih.gov/guidelines/hypertension/child_tbl.htm), computed for age, sex, and height. Hypertension was defined when the SBPi or DBPi was >1.

### Overnight Polysomnography

Overnight polysomnography was conducted and scored using previously-described standard approaches^25^,^26^. An obstructive AHI >2/hrTST along with a nadir SpO2 <92% and/or a respiratory arousal index >2/hrTST served as criteria for OSA diagnosis^26^.

### Neurocognitive assessments

The cognitive tests were administered in the morning, following the sleep study, and consisted of the Differential Ability Scales (DAS)^27^. Children were administered the school age form of the DAS, which yields a Spatial Cluster score in addition to the Verbal, Nonverbal, and global composite score, the latter called the General Conceptual Ability (GCA) score. The sum of the core subtest T-scores is converted to a total battery standard score, the GCA, with a mean of 100 and a standard deviation of 15^28^.

### MRI scanning

We collected brain MRI data from all children within 3–5 days after the sleep study on a Philips Achieva 1.5 Tesla scanner. High-resolution three-dimensional T1-weighted anatomical scans were collected from 16 OSA and 9 control subjects using a custom ultrafast gradient echo “SENSE” sequence (repetition time = 8.16 ms; TE = 3.7 ms; flip angle = 8°; matrix size = 256×256; field of view = 224×224 mm^2^; slice thickness = 1.0 mm; number of slices = 160). Additional control subjects were originally scanned, but were excluded from analysis due to motion artifacts in the images.

We downloaded high-resolution T1-weighted images of remaining control subjects from the NIH Pediatric MRI database (http://pediatricmri.nih.gov/nihpd/info/index.html) with permission. We used a large control cohort, since increasing cohort size is one approach to improve reliability of VBM analyses in OSA^29^. Full details of recruitment and scanning protocols are available from the project website, including confirmation that consent and assent was obtain as appropriate for the age of the subjects, and that procedures were approved by applicable institutional review boards. In brief, the purpose of the Pediatric MRI Study of Normal Brain Development included providing a normative database of the developing brain for comparison with neuroimaging studies of pediatric disease conditions. Participants were recruited from six sites across the United States, and evaluated and screened for health status based on extensive criteria. MRI scans were acquired according to standard protocols, including high resolution T1-weighted anatomical scans at 1 mm isotropic voxel resolution, as used in the present study. Some participants were studied at one or two follow-up visits to obtain longitudinal data. We selected subjects from the database with T1 scans in the age range of our OSA participants. For subjects who fell within the age range during more than one visit, only one recording was used; the choice of which visit was based on ensuring the best age match with the OSA group.

### Analysis

We used SPM12 software for preprocessing and implementation of voxel-based morphometry (VBM), a regional grey matter volume analysis^30^. Each subject’s T1-weighted scan was manually rigid-body realigned to Montreal Neurological Institute (MNI) template space. The manual procedure was also used to check the quality of each T1-weighted scan, and exclude those images with motion artifacts. Scans were corrected for intensity bias due to field inhomogeneities using the SPM “unified” procedure with standard parameter settings^31^. The SPM “DARTEL” procedure^32^ was then used to normalize the T1-weighted scans to the VBM8 template in MNI space, and create regional grey matter maps for each subject. These grey matter maps were “modulated” by the volume change at each voxel, an approach originally termed “optimized” VBM^30^,^33^, and smoothed with a Gaussian filter (full width at half maximum = 10 mm).

The VBM procedure relies on differentiating grey and white matter and other materials present in the head (cerebral spinal fluid [CSF], skull) based on intensity and location. Scans must have distinct intensities for grey and white matter, as well as CSF. The intensity values, or the relative difference between intensities of each tissue type, do not influence the analysis so long as they are distinct. Since T1 weighted anatomical scans by their nature show light intensities for white matter, darker intensities for grey matter, and usually very dark or black intensities for CSF, the specific implementation of the T1 scanning protocol should not influence the results. Thus the VBM procedure is suited to the analysis of scans collected from different scanners and varying protocols, as is the case here.

The average of unsmoothed normalized T1-weighted images was calculated for use as a background for visualizing areas of interest. An average brain mask, consisting of brain regions in normalized space where the probability of grey plus white matter exceeded 0.5, was created for use in the statistical analysis. An estimate of total intracranial volume (TIV) was calculated by voxels where the combined probability of gray matter, white matter and CSF was greater than 0.5. This TIV estimate has only moderate reliability and validity as it is influenced by scanning parameters, specifically the field-of-view, as well as by shape of the base of the skull.

### Statistical modeling

We compared OSA vs. control subjects with an ANCOVA model in SPM12^34^ with sleep status (OSA *vs*. controls) and sex as categorical variables, and age and TIV as continuous variables. Since no differences emerged between control children assessed in the sleep laboratory and database derived controls, all control subjects were combined into a control (non-OSA) group. The brain mask was used to exclude non-brain regions from statistical analysis. Correction for multiple comparisons was performed with family-wise error correction, with threshold at *P* < 0.05. Statistically significant results were visualized by overlaying onto the average of all subjects’ normalized T1-weighted images, and areas of cortical changes were visualized by rendering onto a cortical surface.

To identify any associations between grey matter volume and OSA severity or neurocognitive status, we implemented a regression model in SPM12 with AHI, age and sex as independent variables, and a second model with DAS, age and sex as independent variables. We only assessed grey matter volume relationships in the OSA group, since the pediatric MRI dataset did not include the DAS, and there were few other control subjects. To assess potential scanner-related differences between the Pediatric MRI and OSA data sets, we compared the control data we acquired to those from the database. We used the same model as for the OSA-control comparison, including age and sex as covariates and the brain mask.

## Results

A total of 16 children with OSA and 9 age-, gender-,ethnicity-, and BMI z score-matched controls were evaluated in the sleep laboratory and underwent neurocognitive testing as well as MR imaging procedures at the University of Chicago. Demographic and polysomnographic findings are summarized in Table 1.

**Figure 1:**
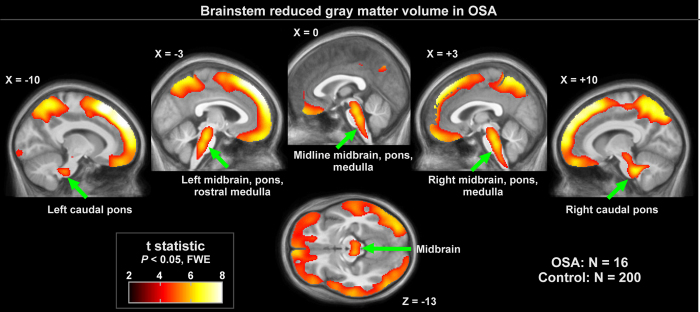
Brainstem regions of significantly lower regional grey matter volume in OSA (n = 16) over control (n = 200) subjects (*P* < 0.01), colored according to significance level. Background is the average of the 216 subjects’ normalized T1-weighted brain scans. Slice locations are in MNI coordinates.

**Figure 2:**
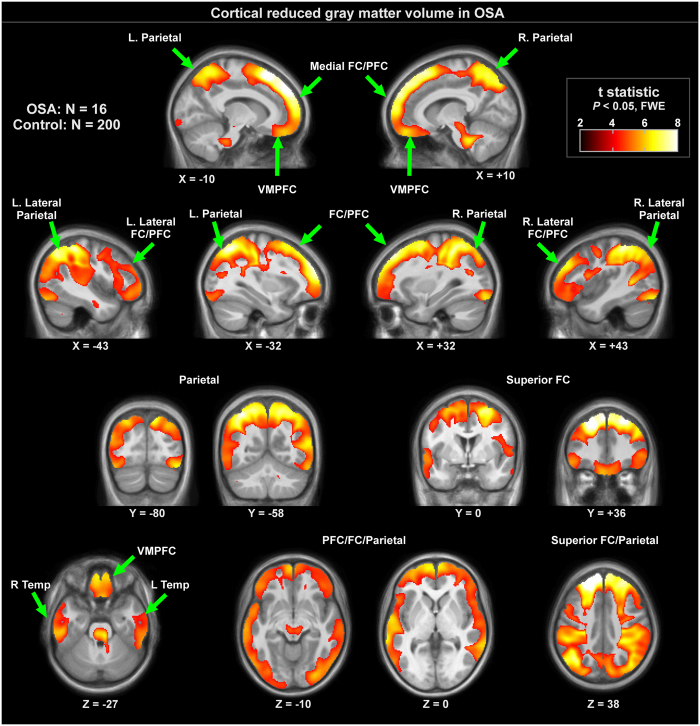
Cortical regions with significantly lower regional grey matter volume in 16 OSA compared to 200 control subjects (*P* < 0.01), colored according to significance level. Background is the average anatomical of 216 subjects. Slice locations are in MNI coordinates. Key: L left, R right, FC frontal cortex, PFC prefrontal cortex, VMPFC ventral medial prefrontal cortex.

**Figure 3:**
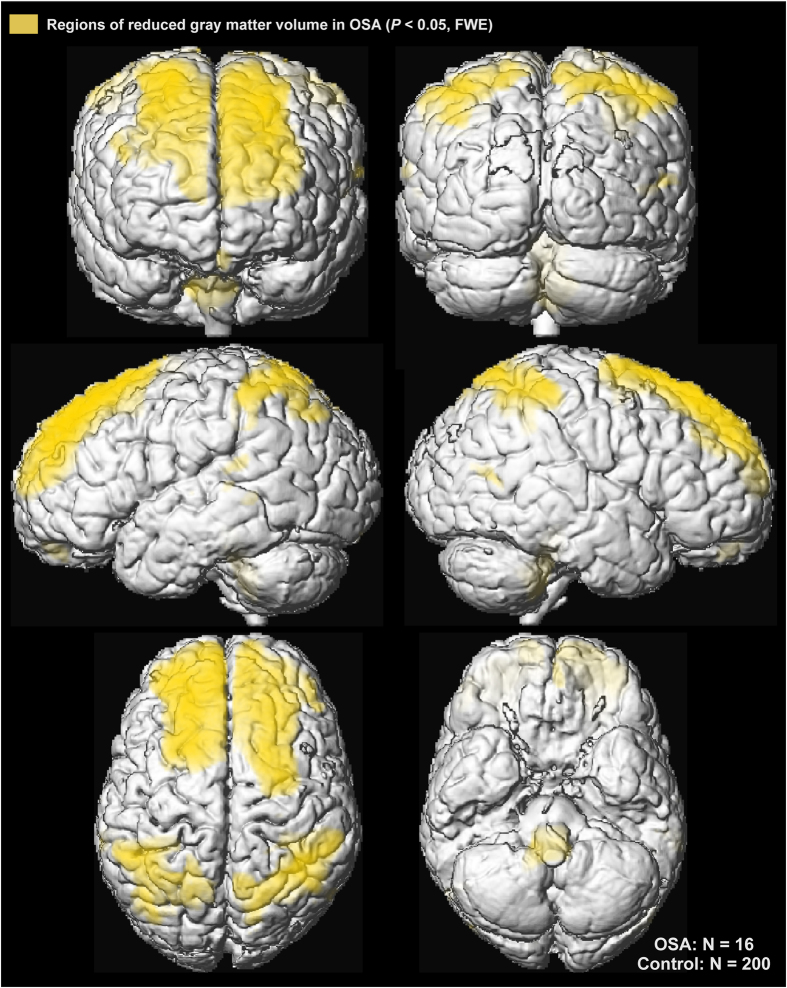
Cortical regions of significantly reduced regional grey matter volume in OSA over control subjects (*P* < 0.01) displayed in yellow on the cortical surface on a single subject in MNI space.

Grey matter volume reductions in OSA subjects appeared throughout extensive areas (35,452 voxels), including the frontal and prefrontal cortices, parietal cortices, temporal lobe, and brainstem (*P* < 0.05, family-wise error correction; Table 2). Brainstem regions are highlighted in Fig. 1, showing volume reductions from the mid-brain through the midline pons and into the medulla. The medial aspects of these three brainstem structures were especially affected. Although the ventral pons did not show changes, volume reduction also appeared in the lateral caudal pons, extending to the right cerebellar peduncles. The majority of cortical regions were affected as well, as shown in Figs 2 and 3. Prefrontal and frontal sites showed volume reductions in all areas, but the bilateral precentral gyrus was not affected. All parietal areas showed reduced volume, but occipital cortices were spared.

There were no significant associations between regional grey matter volumes and severity (as measured with AHI) or DAS scores in OSA subjects, accounting for age, sex and TIV (based on a threshold of *P* = 0.05 with family-wise error correction).

The comparison between our 9 controls and the 191 healthy children from the Pediatric MRI database showed small areas of difference (higher grey matter volume in Pediatric MRI) in the left medial prefrontal and superior-most medial frontal cortices, overlapping 2334 voxels (6.4%) of regions identified as affected in OSA. Of note, Age was not significantly correlated with TIV in either of the 2 groups (OSA: r = −0.08, *P = *0.8; Controls: r = 0.04, *P* = 0.6).

## Discussion

This study shows that in a cohort of 7–11 year-old children clinically and polysomnographically diagnosed with OSA, extensive regionally-demarcated grey matter volume reductions are present. These regional brain changes were not correlated with the severity of respiratory disturbance during sleep and there were no trends indicative of an association with cognitive ability, as determined from the DAS-GCA scores.

Since determining OSA disease duration is not practically possible, subjects may have experienced OSA for several years, and these findings may either reflect delayed neuronal development or disease induced neuronal damage. Similarly, the nature of the grey matter volume changes is unclear; atrophy arising from neuronal damage is one possibility, but reduction in neuronal and glial cell volume could also have occurred, in which case these alterations could be at least partially reversible with treatment. Regardless of the origin of these volume reductions, altered regional grey matter is likely impacting brain functions, and hence cognitive development potential may be at risk in children with OSA.

Before discussing the potential implications of current findings, some methodological points deserve comment. First, sample size enrolled in the present study was relatively small. However, group differences reported herein were of sufficient magnitude and consistency to pass the stringent statistical criteria set forth by the multivariate analyses and post-hoc tests applied. Therefore, the major potential concern that could arise from such small sample size would invoke the possibility for other brain regions that did not emerge as statistically significant in the present study may have emerged if larger samples had been included, as a potential β error cannot be excluded with certainty. Secondly, only one other study has been published in children with OSA using MRI, and their findings are remarkably aligned with the present results^35^. Indeed, Chan and colleagues assessed 23 children with mild (n = 15) and moderate to severe OSA (n = 8) along with 15 control children, and showed regional grey matter losses among the moderate-to-severe OSA group that were circumscribed to only the frontal and temporal gyri^35^. In the present study, all but one of the OSA children suffered from moderate-to-severe OSA, such that the current grey matter reductions concur with those of Chan *et al*^35^. We should further emphasize that the alarming findings indicating grey matter losses in multiple brain regions as reported earlier and in this present study in children are of substantial relevance considering the inability to detect significant improvements in neurocognitive functioning outcomes by the first randomized controlled trial on pediatric OSA and adenotonsillectomy^14^. The discrepancy between cognitive function and MRI grey matter findings further attests to the lack of sensitivity of psychological batteries to identify structural deficits, along with the high degree of variance in the cognitive outcomes associated with pediatric OSA despite the presence of severity-dependent relationships^3^. In addition to our own controls, we included MRI scans from a very large NIH MRI database, which greatly improves the estimation of the true population levels of regional grey matter volumes. One potential consideration is that sleep status was not assessed in this pediatric population, so there may be some subjects with OSA. However, the close to 200 subjects included here would greatly minimized the influence of any unidentified OSA subjects. Furthermore, using such large cohort is a recommendation based on a recent critical review of VBM studies in adult OSA^29^. Of note, different scanners and scanning parameters were used to acquire the brain scans. Since VBM relies predominantly on grey matter, white matter, and CSF intensity levels, scanner variation is unlikely to have influenced these findings. A test between 9 control subjects, scanned with the same conditions as the OSA subjects, and the Pediatric MRI dataset showed minimal variation, although this assessment is limited due to the small number of control scans.

The source of lower regional grey matter volume in OSA subjects cannot be determined with the present data, but several mechanisms can be postulated. In adult OSA, reduced grey matter volume is reflective of atrophy, a process of volume reduction arising from cell death or shrinkage in subjects with long lasting disease condition. Atrophy occurs with normal aging, as well as with disease conditions^36^. However, reduced grey matter volume in a pediatric population could also reflect a delayed or impaired development, since increases in grey matter are seen before 10 years of age^37^. Volume increases occur at a faster rate in 4–8 year period^37^, raising the possibility that OSA at a younger age could impose a greater impact on normal development. The extent and magnitude of regional grey matter changes were surprising even when accounting for the unique neuronal susceptibility to intermittent hypoxia and sleep fragmentation of the developing brain^17^.

The functional consequences of the reduced grey matter volume identified here may account for the typical lower neurocognitive performance in pediatric OSA^38^. While the present data did not show significant associations between the MRI findings and a measure of cognitive performance (i.e., DAS score), higher grey matter volume has been associated with higher intelligence quotients (IQ), although only in an older age pediatric group (mean age 15.4 years vs groups with mean ages of 7.6 and 10.9 years). This IQ and grey matter volume association may therefore not be discernible in children in the age range of the current sample. Brainstem volume reductions have been associated with autism^39^, but volume in this region is not associated with IQ^40^.

The neurocognitive consequences of OSA in children have been extensively evaluated with divergent findings, whereby at any level of OSA severity, not every child will necessarily manifest the presence of cognitive deficits^38^. These findings have prompted the assumption that both genetic and environmental factors contribute to this variance^41^. In the largest cohort to date, we have recently shown that as the severity of OSA increases, a dose-dependent increase in the probability of reductions in cognitive performance can be detected, whereby AHI >5/hrTST was associated with a mean of 5 DAS score points reduction (95% CI: 0–8.5; n = 1,110 children)^3^. Thus, in the context of a standard deviation of 10 points for DAS scores^28^, very large sample sizes would be required to ascertain the presence or absence of an association between cognition and grey matter changes. Furthermore, since most children with OSA appear to functionally compensate, at least to a certain extent for the neural deficits imposed by the disease^15^, further variance would be introduced into the exploration of DAS-grey matter associations, thereby contributing to the lack of statistical significance. Notwithstanding, the close agreement between the regional grey matter losses in the present study and those reported by Chan *et al*^35^. would signify that an AHI >5/hrTST may reflect a cut-off above which anatomical evidence will emerge and attest to neuronal cell losses among children suffering from OSA. Relatedly, the lack of association between grey matter volume and AHI or neurocognitive status was performed with a much lower sensitivity compared with the OSA-control comparison, due to the smaller number of subjects; it is still possible that a small or moderate relationship between OSA severity and cognitive function is present. Alternatively, large scale longitudinal prospective studies aimed at examining changes in grey matter and cognition before and following treatment in those children with pre-treatment evidence of OSA and cognitive deficits should shed some insights into aforementioned considerations.

The present findings show both similarities and differences compared to adult findings of grey matter volume changes in OSA, a finding that was not unexpected given the wide variation in results reported^29^. Before we discuss these issues, we should first emphasize that the moderate to severe magnitude of OSA in our current pediatric cohort would constitute the equivalent of either mild or normal sleep studies in adult OSA, since the current clinical polysomnographic criteria for diagnosis of OSA markedly differ in these 2 populations. Nonetheless, volume reductions in the middle temporal lobes, as found here, were also observed in several adult OSA studies^42^. Adult OSA patients also show superior frontal and parietal cortex volume reductions^20^,^24^, although in isolated areas, as opposed to the entire extent of brain structures in pediatric condition. Cerebellar grey matter reductions, especially in the cortex, are also evident in adults^20^, and in the present study pediatric OSA showed effects in a region extending from the brainstem towards cerebellar peduncles, but not cerebellar cortex. One notable contrast with adult findings is the lack of sub-cortical limbic regions affected in this pediatric OSA study, such as the hippocampus and adjacent grey matter^20^,^24^. A second contrast is the lack of grey matter alterations in brainstem regions of adult OSA patients. These differences may reflect unique age-, duration-, or severity-related differential regional susceptibilities to the underlying OSA. Finally, several studies in adults have reported improvements in MRI findings following treatment with continuous positive pressure (CPAP) ventilation via a mask^23^,^24^.

In summary, children with moderate to severe OSA exhibit extensive regionally demarcated grey matter losses compared to healthy children. In the contextual setting that OSA is fraught with increased risks for a variety of end-organ morbidities, the mechanisms underlying such extensive MRI changes, the exact nature of the grey matter reductions and their potential reversibility remain virtually unexplored, and should prompt intensive future research efforts in this direction.

## Additional Information

**How to cite this article:** Philby, M. F. *et al*. Reduced Regional Grey Matter Volumes in Pediatric Obstructive Sleep Apnea. *Sci. Rep.* **7**, 44566; doi: 10.1038/srep44566 (2017).

**Publisher's note:** Springer Nature remains neutral with regard to jurisdictional claims in published maps and institutional affiliations.

## Figures and Tables

**Table 1:** General characteristics of children with OSA and healthy controls.

	OSA (n = 16)	Control (n = 9)
Age (years)	6.9 ± 2.8	6.8 ± 2.2
Gender (male, %)	59.5	55.5
Ethnicity		
Caucasian (%)	41.8	45.4
African-American (%)	50.0	52.3
Other	8.2	2.3
BMI Z score	1.28 ± 0.22	1.08 ± 0.16*
Systolic blood pressure (mmHg)	108.0 ± 10.1**	102.3 ± 8.4*
SBPi	0.93 ± 0.08**	0.87 ± 0.07*
Diastolic blood pressure (mmHg)	66.9 ± 7.3	63.4 ± 6.7*
DBPi	0.92 ± 0.08	0.81 ± 0.06*
Obstructive AHI (events/hour)*	14.6 ± 12.4^§^	0.4 ± 0.2
	11.7 (9.85)	0.3 (0.1)
S_p_O_2_ Nadir (%)	76.7 ± 11.6^§^	94.1 ± 2.9
ODI3% (/hrTST)	11.6 ± 10.6^§^	0.2 ± 0.2
Total Arousal Index (/hrTST)	17.2 ± 5.2^§^	7.3 ± 2.8
DAS General Conceptual Ability Score*	89.6 ± 8.0	103.8 ± 3.2
	87.5 (11.5)	105 (4)

Abbreviations: AHI – apnea-hypopnea index; BMI body mass index; OB – obese; ODI3% - oxygen desaturation index 3%; OSA – obstructive sleep apnea; RAI – respiratory arousal index.

Data are presented as mean ± SD. *Data are not normally distributed, median and interquartile ranges (IQR) are also provided.

**Table 2:** Statistical results for gray matter volume reductions in OSA vs. controls as shown by SPM.

Cluster #	Cluster		Peak		
	P (FWE)	voxels	*P* (FWE)	T	x,y,z (mm)
1	0	10098	0	7.73	−27 50 30
			0	7.49	−23 33 45
			0	7.03	−6 48 41
2	0	8845	0	7.29	20 17 56
			0	6.99	27 39 44
			0	6.76	26 30 51
3	0	6758	0	6.81	−36 −66 51
			0	6.08	−44 −41 51
			0	5.68	−20 −57 57
4	0	7483	0	6.14	41 −41 53
			0	5.91	27–47 53
			0	5.75	50–30 47
5	0	1601	0.001	5.48	8 −35 −35
			0.001	5.41	−2 −27 −24
6	0.011	138	0.014	4.77	44 −81 −18
			0.023	4.63	54 −65 −20
7	0.004	291	0.016	4.72	56 −60 14
			0.021	4.65	41 −50 5
8	0.038	11	0.018	4.7	−51 −71 −18
9	0.017	82	0.019	4.68	−62 −30 15
10	0.03	26	0.023	4.64	−1 0 5
11	0.015	92	0.028	4.58	−2 53 −26
12	0.042	5	0.03	4.56	26–17 45
13	0.033	20	0.033	4.53	−56–26 5
14	0.047	1	0.048	4.43	−68 −35 −2
15	0.047	1	0.05	4.42	−62 −9 2

Each cluster has cluster-level *P*-value (FWE corrected) and number of voxels indicated. For each cluster one or more major peaks show p value (FWE corrected),t statistic, and location in template space. Other SPM results include: height threshold t = 4.42 (for *P* < 0.05, FWE); expected voxels per cluster = 92.4; expected number of clusters = 0.05; degrees of freedom = [1,211]; effective FWHM = 19.9 20.1 19.5 mm; voxel size = 1.5 mm.
